# Myeloid-Specific Deletion of Diacylglycerol Lipase α Inhibits Atherogenesis in ApoE-Deficient Mice

**DOI:** 10.1371/journal.pone.0146267

**Published:** 2016-01-05

**Authors:** Julian Jehle, Friedrich Felix Hoyer, Benedikt Schöne, Philipp Pfeifer, Katharina Schild, Imke Jenniches, Laura Bindila, Beat Lutz, Dieter Lütjohann, Andreas Zimmer, Georg Nickenig

**Affiliations:** 1 Klinik II für Innere Medizin, Universität Bonn, Bonn, Germany; 2 Massachusetts General Hospital, Center for Systems Biology, Boston, United States of America; 3 Institut für Molekulare Psychiatrie, Universität Bonn, Bonn, Germany; 4 Institut für Physiologische Chemie, Johannes Gutenberg Universität Mainz, Mainz, Germany; 5 Institut für Klinische Chemie und Klinische Pharmakologie, Universität Bonn, Bonn, Germany; Nagoya University, JAPAN

## Abstract

**Background:**

The endocannabinoid 2-arachidonoylglycerol (2-AG) is a known modulator of inflammation. Despite its high concentration in vascular tissue, the role of 2-AG in atherogenesis has not yet been examined.

**Methods:**

ApoE-deficient mice were sublethally irradiated and reconstituted with bone marrow from mice with a myeloid-specific knockout of the 2-AG synthesising enzyme diacylglycerol lipase α (Dagla) or control bone marrow with an intact 2-AG biosynthesis. After a cholesterol-rich diet for 8 weeks, plaque size and plaque morphology were examined in chimeric mice. Circulating inflammatory cells were assessed by flow cytometry. Aortic tissue and plasma levels of endocannabinoids were measured using liquid chromatography-multiple reaction monitoring.

**Results:**

Mice with Dagla-deficient bone marrow and circulating myeloid cells showed a significantly reduced plaque burden compared to controls. The reduction in plaque size was accompanied by a significantly diminished accumulation of both neutrophil granulocytes and macrophages in atherosclerotic lesions of Dagla-deficient mice. Moreover, CB2 expression and the amount of oxidised LDL within atherosclerotic lesions was significantly reduced. FACS analyses revealed that levels of circulating inflammatory cells were unaltered in Dagla-deficient mice.

**Conclusions:**

Myeloid synthesis of the endocannabinoid 2-AG appears to promote vascular inflammation and atherogenesis. Thus, myeloid-specific disruption of 2-AG synthesis may represent a potential novel therapeutic strategy against atherosclerosis.

## Introduction

Atherosclerosis and its clinical manifestations coronary, peripheral and carotid artery disease are the number one cause of death worldwide [1]. Given the increasing prevalence of these diseases and the limited treatment options, the development of novel therapeutic strategies against atherosclerosis represents an unmet medical need. Atherosclerosis is a chronic inflammatory disease and cells of both the adaptive and innate immune system are involved in the initiation and propagation of atherosclerotic lesions (reviewed in [2]). The endocannabinoid system has been shown to modulate inflammatory processes in various models of inflammatory diseases [3–8]. The endocannabinoid system mainly consists of the cannabinoid receptors CB1 and CB2, their endogenous ligands, which are known as endocannabinoids, and their respective synthesising and degrading enzymes [9–11]. The two best-studied endocannabinoids are N-arachidonoylethanolamide (anandamide, AEA) and 2-arachidonoylglycerol (2-AG), the latter being synthesised by diacylglycerol lipase (DAGL) and degraded by monoacylglycerol lipase (MAGL; [12]). While the relevance of AEA and its regulating enzymes to atherogenesis has already been demonstrated before [6; 13], the significance of 2-AG to the formation of atherosclerosis is less well established. Montecucco and coworkers have already described elevated 2-AG levels in the aortas of ApoE-deficient mice following a high cholesterol diet [14]. Moreover, their in vitro data support the hypothesis that 2-AG might enhance atherogenesis by attracting monocytes to the site of inflammation. However, this study does not provide evidence for a causal connection between elevated 2-AG levels and increased vascular inflammation in vivo. Interestingly, both 2-AG and AEA plasma levels are significantly elevated in patients suffering from coronary artery disease by almost two-fold compared to patients without coronary artery disease [15]. Furthermore, plasma and aortic tissue levels of 2-AG exceed those of AEA by 100-fold in atherosclerotic mice [5]. Since the endocannabinoid system is abundantly expressed in myeloid cells, and since these cells contribute to vascular inflammation and atherogenesis, we generated a chimeric mouse model of an atherosclerosis prone ApoE^-/-^ mouse, whose bone marrow exhibits a myeloid specific deletion of 2-AG synthesising enzyme diacylglycerol lipase α (DAGL-α, LysM^wt/cre^ Dagla^fl/fl^). We hypothesised, that 2-AG is an important regulator of atherogenesis, and that deletion of Dagla in myeloid cells affects atherosclerotic lesion formation.

## Methods

ApoE^-/-^ mice (C57BL/6J genetic background; Charles River, Wilmington, USA) were sublethally irradiated with 9.25 Gy and their bone marrow was reconstituted with bone marrow of either LysM^wt/cre^ Dagla^fl/fl^ mice or control bone marrow from LysM^wt/wt^ Dagla^fl/fl^ mice (both C57BL/6J genetic background, [16]). Mice were kept at 22°C room temperature and at a 12-hour dark-light-cycle. Drinking water and rodent chow were available *ad libitum*. Laboratory animal use was in accordance with institutional guidelines and German animal protection law. Approval was granted by the North Rhine Westphalian State Agency for Nature, Environment and Consumer Protection (reference number 84–02.04.2014.A419) which considered ethics and welfare aspects of all animals protocols in their approval. Furthermore, animal protocols were submitted to and approved by the local ethics committee of the University of Bonn.

One week after successful reconstitution, chimeric mice were fed a high fat and cholesterol rich diet for 8 weeks containing 21% fat, 19.5% casein, and 1.25% cholesterol (Ssniff, Soest, Germany). Afterwards, mice were sacrificed by cervical dislocation under 5% isoflurane (Abbott Laboratories, Chicago, USA) anesthesia and hearts were excised for plaque size- and plaque morphology analyses. Aortic tissue was used for reactive oxygen species measurements. Innate and adaptive immune response were examined using flow cytometry of blood and bone marrow. Endocannabinoids (eCBs) were quantitatively determined by mass spectrometry (MS) using liquid chomatography-multiple reaction monitoring (LC-MRM) in plasma and aortic tissue. Plasma cholesterol levels were analysed using gas chromatography–flame ionisation detection, as recently described [5].

### 2.1 Culture of primary macrophages and Real-Time PCR

Bone marrow cells of Dagla-deficient LysM^wt/cre^ Dagla^fl/fl^ mice and of control mice with an unimpaired 2-AG biosynthesis were flushed out of mice’ femur and tibia with PBS and filtered using a 70 μm cell strainer (BD Biosciences, San Jose, USA). Cells were subsequently incubated in 30% L-cell supernatant, containing M-CSF for 6 days which caused bone marrow cells to differentiate into primary macrophages. Medium was changed every 48 hours. Confluent cells were washed once with PBS, harvested with 1 ml Trizol® reagent (Ambion life technologies / Thermo Fisher Scientific Inc., Waltham, USA) and transferred into a 1.5 ml reaction tube. Hereafter, tubes were centrifuged at 18,000 x g and 4°C for 15 minutes and the upper phase containing the RNA was transferred into a new reaction tube. RNA was precipitated by adding identical volumes of isopropanol. After 1 hour, precipitated RNA was washed twice with 75% ethanol and finally resuspended in RNAse free H_2_O. The RNA content was measured using a Nanodrop 2000c spectrophotometer (PeqLab Biotechnologie GmbH, Erlangen, Germany). 2 μg of RNA were reversely transcribed into cDNA using the Omniscript RT kit (Qiagen GmbH, Hilden, Germany) according to the manufacturer’s instructions. cDNA was diluted and pipetted onto a 96-well PCR plate in triplets. Dagla and Daglb-specific Taqman probes and the apporpriate master mix (all by Thermo Fisher Scientific Inc.) were added into the wells. Samples were analysed using a 7500 Fast Real-Time PCR system and 7500 software v.2.0.6 (both Thermo Fisher Scientific Inc.).

In order to further expand our view on the regulation of Dagla and Daglb within the atherosclerotic vessel wall, we performed quantitative Fast Real-Time PCR analyses on lysed aortic tissue of mice from both groups. Tissue samples of the abdominal aorta of ApoE-deficient mice with Dagla-deficient myeloid cells and ApoE-deficient controls with Dagla-competent myeloid cells were lysed in Trizol® reagent using a Schuett homogen plus tissue lyser (Schuett-biotec GmbH, Göttingen, Germany). RNA was extracted as described above.

### 2.2. Blood pressure, heart rate

Blood pressure and heart rate were assessed non-invasively using a Volume Pressure Recording system (Kent Scientific Corporation, Torrington, USA). Mice were accustomed to the tail cuff device for 20 minutes on three consecutive days. Afterwards, systolic blood pressure and heart rate were measured on three consecutive days.

### 2.3. Superoxide release

Superoxide content in aortic tissue was assessed by L012 chemiluminescence as described before (Hoyer et al., 2011). Aortic tissue was transferred into chilled modified Krebs-HEPES buffer containing, in mM: NaCl 99.01, KCl 4.69, CaCl_2_ 1.87, MgSO_4_ 1.20, Na HEPES 20.0, K_2_HPO_4_ 1.03, NaHCO_3_ 25.0, D(+)glucose 11.1, adjusted to pH 7.40. Then, aortic tissue was incubated in Krebs-HEPES buffer with 100 μM L012 for 5 minutes. Hereafter, chemiluminescence was measured over 10 minutes in a scintillation counter (Lumat LB 9501, Berthold, Wildbad, Germany) at 1-minute intervals, and the 10-minute value was used for analysis.

### 2.4. Plaque-size and plaque-morphology analyses

Hearts of chimeric ApoE^-/-^ LysM^wt/cre^ Dagla ^fl/fl^ mice and controls were excised, embedded in tissue freezing medium (Leica Biosystems GmbH, Wetzlar, Germany) and snap frozen at -80°C. Frozen sections through the aortic sinus were obtained using a Leica CM 1900 cryostat (Leica Biosystems GmbH, Wetzlar, Germany).

Plaque size was assessed following oil red staining (Oil red O, Sigma-Aldrich, St. Louis, USA). In brief, slides were fixed in 4% paraformaldehyde for 45 minutes before being rinsed in 60% isopropanol. Hereafter, slides were stained using oil red O and haematoxylin.

Neutrophil invasion into the vessel wall was measured by Ly6G (primary antibody: Ly6G clone 1A8, BD biosciences; secondary antibody: Cy3 AffiniPure Donkey anti-Rat IgG, Jackson ImmunoResearch Laboratories, Inc., West Grove, USA) staining. Slides were fixed in acetone at -20°C for 30 minutes before being exposed to 1% H_2_O_2_ in methanol for 20 minutes. Subsequently, slides were blocked in 10% normal goat serum in PBS before the primary antibody, which had been diluted by 1:200 in NGS, was pipetted onto the slides and incubated at 4°C over night. On the following day, slides were rinsed in PBS and the secondary antibody, which had been diluted by 1:500 in PBS, was administered for 1 hour at room temperature in the dark. Hereafter, slides were fixed and nuclei were stained using Vectashield mounting medium with DAPI (Vector Laboratories, Inc., Burlingame, USA).

Macrophage accumulation was detected by staining CD68 (primary antibody: α-CD68 rat IgG2a, Acris antibodies GmbH, Herford, Germany; secondary antibody: Cy3 AffiniPure Donkey anti-Rat IgG, Jackson ImmunoResearch Laboratories, Inc.). Slides were fixed in acetone at -20°C for 20 minutes, then washed in PBS and blocked in 1% BSA in PBS for 30 minutes. Afterwards, sections were incubated at 4°C over night with the primary antibody, which had been diluted by 1:100 in blocking solution. On the following day, slides were washed with PBS and the secondary antibody, which had been diluted by 1:500 in PBS, was administered at room temperature in the dark for 1 hour. Afterwards, slides were fixed and nuclei were stained using Vectashield mounting medium with DAPI (Vector Laboratories).

Collagen fibres were stained by Picro Sirius staining (Direct Red 80, Sigma-Aldrich). Slides were fixed in ethanol, then rinsed in deionised water and stained with haematoxylin and Direct Red for 15 minutes. Afterwards, slides were fixed in ethanol and xylene.

Oxidised LDL (oxLDL) formation was examined in frozen sections through the aortic sinus. Briefly, slides were fixed in acetone at -20°C for 20 minutes, then washed in PBS and blocked in 1% BSA in PBS for 30 minutes. Afterwards, sections were incubated at 4°C over night with the primary antibody (rabbit polyclonal antidody to oxLDL, Bioss Inc., Woburn, USA) which had been diluted by 1:100 in blocking solution. On the following day, slides were washed with PBS and the secondary antibody (goat anti rabbit Cy3, Dianova GmbH, Hamburg, Germany) which had been diluted by 1:500 in PBS containing 1% BSA, was administered at room temperature in the dark for 1 hour. Afterwards, slides were fixed and nuclei were stained using Vectashield mounting medium with DAPI (Vector Laboratories).

CB2 expression was studied using the rabbit polyclonal antidody to Cannabinoid Receptor II (Abcam plc., Cambridge, UK) primary antibody and a Cy3 conjugated goat anti rabbit secondary antibody. Following fixation in 4% paraformaldehyde and blocking with PBS containing 1% BSA and 10% NGS, slides were exposed to the primary antibody for 22 hours, Hereafter, slides were washed and incubated with the secondary antibody which had been diluted by 1:250 in PBS containing 0.5% BSA for 2 hours at room temperature. Finally, nuclei were stained using Vectashield mounting medium with DAPI (Vector Laboratories).

CB1 expression was studied using the rabbit polyclonal antidody to Cannabinoid Receptor I (Abcam plc., Cambridge, UK) and a Cy3 conjugated goat anti rabbit secondary antibody. Coexpression of CD68 was evaluated by subsequential costaining using a CD68 primary antibody (α-CD68 rat IgG2a, Acris antibodies GmbH, Herford, Germany) and a Cy2-conjugated goat-anti-rabbit secondary antibody (Dianova GmbH). Following fixation in 4% paraformaldehyde and blocking with PBS containing 2% BSA, 2% NGS and 0.1% Triton X-100 (Sigma-Aldrich) slides were exposed to the primary antibody for 24 hours, Hereafter, slides were washed and incubated with the secondary antibody which had been diluted by 1:500 in PBS containing 0.5% BSA for 2 hours at room temperature. Hereafter, CD68 staining was performed as described, but with the above mentioned Cy2 conjugated secondary antibody. Finally, nuclei were stained using Vectashield mounting medium with DAPI (Vector Laboratories).

CD3 expression was examined in frozen sections. Following fixation in acetone, slides were blocked in 1% BSA in PBS for 30 minutes. Afterwards, sections were incubated at 4°C over night with the primary antibody (rabbit polyclonal antidody to CD3, Abcam plc.) which had been diluted by 1:100 in blocking solution. On the following day, slides were washed with PBS and 1:500 secondary antibody (Cy3 conjugated goat anti rabbit, Dianova GmbH) was administered at room temperature in the dark for 1 hour. Lastly, slides were fixed and nuclei were stained using Vectashield mounting medium with DAPI (Vector Laboratories).

Microphotographic pictures were taken using a Zeiss Axiovert 200M microscope (Carl Zeiss Jena GmbH, Jena, Germany) and Axiovision 4.8 software (Carl Zeiss Jena GmbH). Group affiliation (Dagla-deficient mice versus controls) of each picture was blinded prior to assessment. Positively stained areas were normalised to the size of the vessel wall. The size of the vessel wall on immunofluorescence staining images was measured using the DAPI chanel by comparing nuclei distribution patterns to the haematoxylin stained neighbouring sections of identical hearts on oil red stained slides.

### 2.5. Flow cytometry analyses of circulating immune cells

Blood samples were drawn from sacrificed mice for flow cytometry analyses (FACSCalibur; BD biosciences). Leukocytes were stained for CD3 (FITC-conjugated), CD19 (PE-conjugated), and CD11b (APC-conjugated; clones 17A2, 1D3 and M1/17, BD biosciences) or CD11b (PE-conjugated), Ly6C (FITC-conjugated), and Ly6G (APC-conjugated; clones M1/17, RB6-8C5, AL-21, BD biosciences) respectively. Prevalence of these surface markers within a specified leukocyte gate was determined after measuring 100,000 counts during FACS analysis. Unstained samples and isotype identical antibodies were used as controls. Data acquisition and analysis was performed using Cell Quest software (BD biosciences).

### 2.6. Assessment of inflammatory cytokines

Inflammation related cytokines were examined in plasma samples of ApoE-deficient mice with Dagla-deficient myeloid cells and ApoE-deficient controls with Dagla-competent myeloid cells. MCP-1-, IL-10-, and IFN-γ- ELISA kits (enzyme-linked immunosorbent assay; all by Abcam plc.) were employed according to the maufacturer’s instructions. Plasma samples were diluted by 1: 3.5 with dilution buffer supplied with the ELISA kits.

### 2.7. Extraction of endocannabinoids and LC-MRM quantification

Concentrations of anandamide (AEA), 2-arachidonoyl glycerol (2-AG), oleylethanolamide (OEA), palmitoylethanolamide (PEA), and arachidonic acid (AA) were quantified in plasma and aortic tissue samples by liquid chromatography-multiple reaction monitoring, using LC-MRM conditions as described [17]. For extraction of endocannobinoids, the frozen aortic tissue samples were transferred in pre-cooled extraction tubes containing cold steel balls. The samples were then spiked with 50 μl solution of deuterated eCBs in acetonitrile, as internal standards, followed by the addition of 250 μl 0.1 M formic acid, which served as homogenisation buffer and 300 μl ethylacetate/n-hexane (9:1, v/v) as extraction solvent. The samples were homogenised for 40 seconds at 30 Hz using a tissue lyser. (Qiagen GmbH). The homogenates were then centrifuged for 10 minutes at 5000 x g at 4°C and then kept at -20°C for 20 minutes to freeze the aqueous phase. The organic phase was recovered in 96 well plates, evaporated under a gentle stream of nitrogen and reconstituted in 50 μl acetonitrile/water (1:1, v/v) using an automated pipetting machine. The samples were then subjected to LC-MRM analysis. The aqueous phase was used for protein content determination using a BCA assay. For plasma eCBs extraction, the plasma samples were first thawed at 4°C, and then 50 μl spike solution of internal standards in acetonitrile and 250 μl ethylacetate: n-hexane (9:1, v/v) were added to the samples. The samples were vortexed for 30 seconds and then centrifuged for 10 minutes at 16000 g and 4°C. The samples were then kept at -20°C for 10 min, the upper organic phase was recovered in 96 well plates, evaporated to dryness and the extract reconstituted in 50 μl acetonitrile/water (1:1, v/v) for LC-MRM analysis. Throughout the eCB extraction procedure and intermediate steps, the samples and tubes were invariably kept on ice to prevent artifical alteration in the endogeneous levels of the eCBs originating from enzymatic or chemical degradation and/or ex-vivo synthesis of eCBs.

The amounts of internal standards used for aorta and plasma eCBs extraction, and the calibration curve ranges were tailored using standard aorta and plasma samples. The eCBs values were normalised to the protein content, in case of aorta, and to the plasma volume used for plasma samples.

### 2.8. Statistical analyses

Data are presented as mean ± SEM. Statistical differences of continuous variables between two groups were determined with Microsoft excel software (Microsoft, Redmond, USA) and Origin 8.0 software (OriginLab Corporation, Northampton, USA) using unpaired Student's *t*-tests (two-sided tests). For the comparison of three groups, one-way ANOVA with subsequent pairwise Student's two sided *t*-tests was applied. *P* < 0.05 was considered statistically significant.

## Results

### 3.1. Deletion of the Dagla gene

Successful deletion of the Dagla gene was confirmed by Real-Time PCR from primary macrophages. Macrophages from LysM^wt/cre^ Dagla^fl/fl^ mice showed a highly significant reduction in Dagla mRNA expression by 83.7% (0.163 ± 0.031; n = 3–4; p = 0.0004). Simultaneously, we performed a quantitative Fast Real-Time PCR assay employing a TaqMan probe specific for Daglb. Daglb mRNA levels were unaltered in Dagla-deficient macrophages compared to controls (1.10 ± 0.20-fold; n = 3–4; p = 0.78).

In aortic tissue, neither Dagla nor Daglb mRNA expression levels differed between the two groups. In aortic tissue of LysM^wt/cre^ Dagla^fl/fl^ mice Dagla mRNA expression was 0.91 ± 0.20-fold compared to control (n = 10; p = 0.78), and Daglb mRNA expression was 1.23 ± 0.42-fold compared to control (n = 10; p = 0.68). These data suggest that the contribution of Dagla mRNA levels in myeloid cells to total Dagla mRNA levels in aortic tissue is limited and that myeloid Dagla deficiency does not alter overall Daglb mRNA levels in aortic tissue.

### 3.2. Body weight, heart rate, blood pressure, cholesterol

Mice were sublethally irradiated. One group was reconstituted with bone marrow bearing a myeloid specific deletion of Dagla, the other group received control bone marrow with an intact 2-AG biosynthesis. Following bone marrow transplantation, chimeric mice were fed a high-cholesterol diet for 8 weeks. Hereafter, mice were sacrificed and atherosclerotic plaque burden and plaque morphology were assessed in both groups. Body weight, heart rate, systolic blood pressure and serum cholesterol levels are depicted in Table 1. There were no statistically significant differences between the two groups.

**Table 1:** Heart rate, blood pressure, cholesterol, weight.

	Control	LysM^cre^ Dagla^fl/fl^	p
Heart rate [bpm]	722.24 ± 20.54	727.38 ± 23.07	0.8702
Systolic blood pressure [mmHg]	155.54 ± 6.63	155.78 ± 4.19	0.9757
Plasma cholesterol [mg/dl]	307.50 ± 24.10	286.00 ± 58.72	0.5177
Weight [g]	25.36 ± 1.49	24.82 ± 1.10	0.7863

Heart rate, blood pressure, and weight were measured before mice were sacrificed. Serum cholesterol levels were measured using gas chromatography–flame ionisation detection. Data are presented as mean ± standard error of the mean; n = 10. Cre, cyclisation recombination; Dagla, diacylglycerol lipase α; fl, flanked by loxP.

### 3.3. Reactive oxygen species

Reactive oxygen species (ROS) formation was measured by L012 chemiluminescence in aortic tissue and normalised to tissue weight. No differences were seen between the two groups (191.7 ± 31.8 RLU/s/mg vs. 204.1 ± 41.3 RLU/s/mg, n = 10, p = 0.81).

### 3.4. Atherosclerotic plaque burden and plaque morphology

**Fig 1:**
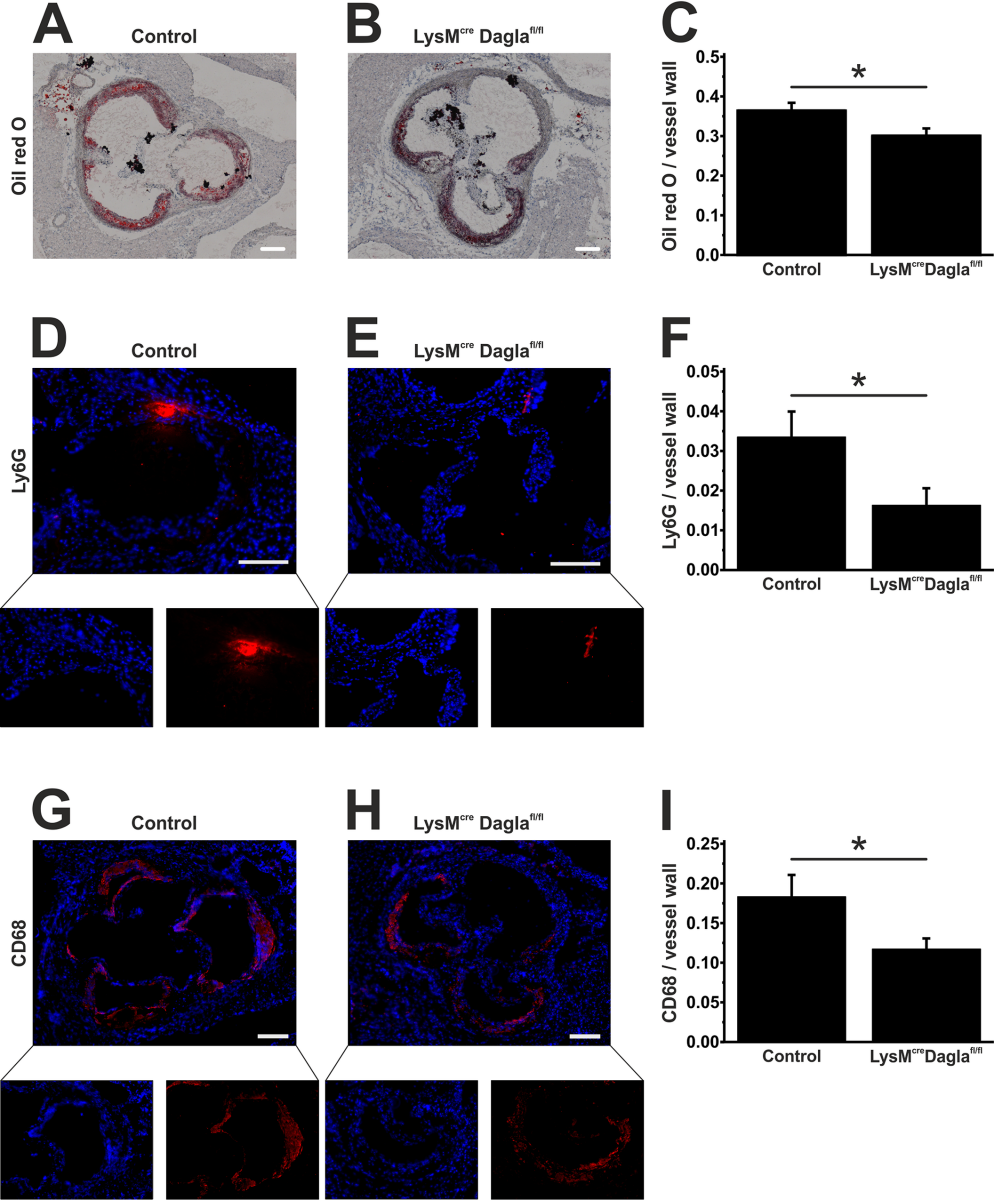
Assessment of plaque burden and plaque morphology. Frozen sections through the aortic sinus of both control mice and Dagla-deficient mice were stained using oil red O to visualise atherosclerotic plaques (A, B). Cumulative plaque area was divided by the size of the vessel wall to obtain relative plaque burden (C). Neutrophil granulocytes were identified by staining Ly6G (D, E). Ly6G positive areas were normalised to the size of the vessel wall (F). Macrophages were identified by staining CD68 (G, H). CD68 positive areas were normalised to the size of the vessel wall (I). Data are presented as mean ± standard error of the mean; n = 9–13; *, p < 0.05. Scale bar, 200 μm. CD, cluster of differentiation; cre, cyclisation recombination; Dagla, diacylglycerol lipase α; fl, flanked by loxP; Ly6G, lymphocyte antigen 6G; LysM, lysozyme 2 gene.

Atherosclerotic plaques were stained in frozen sections through the aortic sinus of chimeric ApoE^-/-^ LysM^wt/cre^ Dagla^fl/fl^ mice and controls by oil red O staining. Plaqe size was measured and normalised to the size of the vessel wall. Dagla-deficient ApoE^-/-^ LysM^wt/cre^ Dagla^fl/fl^ mice showed a significantly reduced plaque burden compared to control mice as depicted in Fig 1A–1C (0.36 ± 0.02 vs. 0.30 ± 0.02, n = 13, p = 0.03).

We next quantified neutrophil granulocytes invading the vessel wall. Thus, we stained frozen sections of both groups for Ly6G, which is a GPI-anchored protein expressed on neutrophils. As shown in Fig 1D–1F, control mice displayed a robust infiltration by neutrophils, predominantly at the marginal regions of atherosclerotic plaques, which was significantly diminished in Dagla-deficient mice (0.033 ± 0.007 vs. 0.016 ± 0.004, n = 9, p = 0.047).

In order to identify macrophages in atherosclerotic lesions, we stained frozen sections through the aortic sinus for CD68 (Fig 1G–1I). In accordance with the results for Ly6G staining, Dagla-deficient mice showed a significantly reduced infiltration of macrophages of the vessel wall (0.18 ± 0.03 vs. 0.12 ± 0.01, n = 10, p = 0.04).

**Fig 2:**
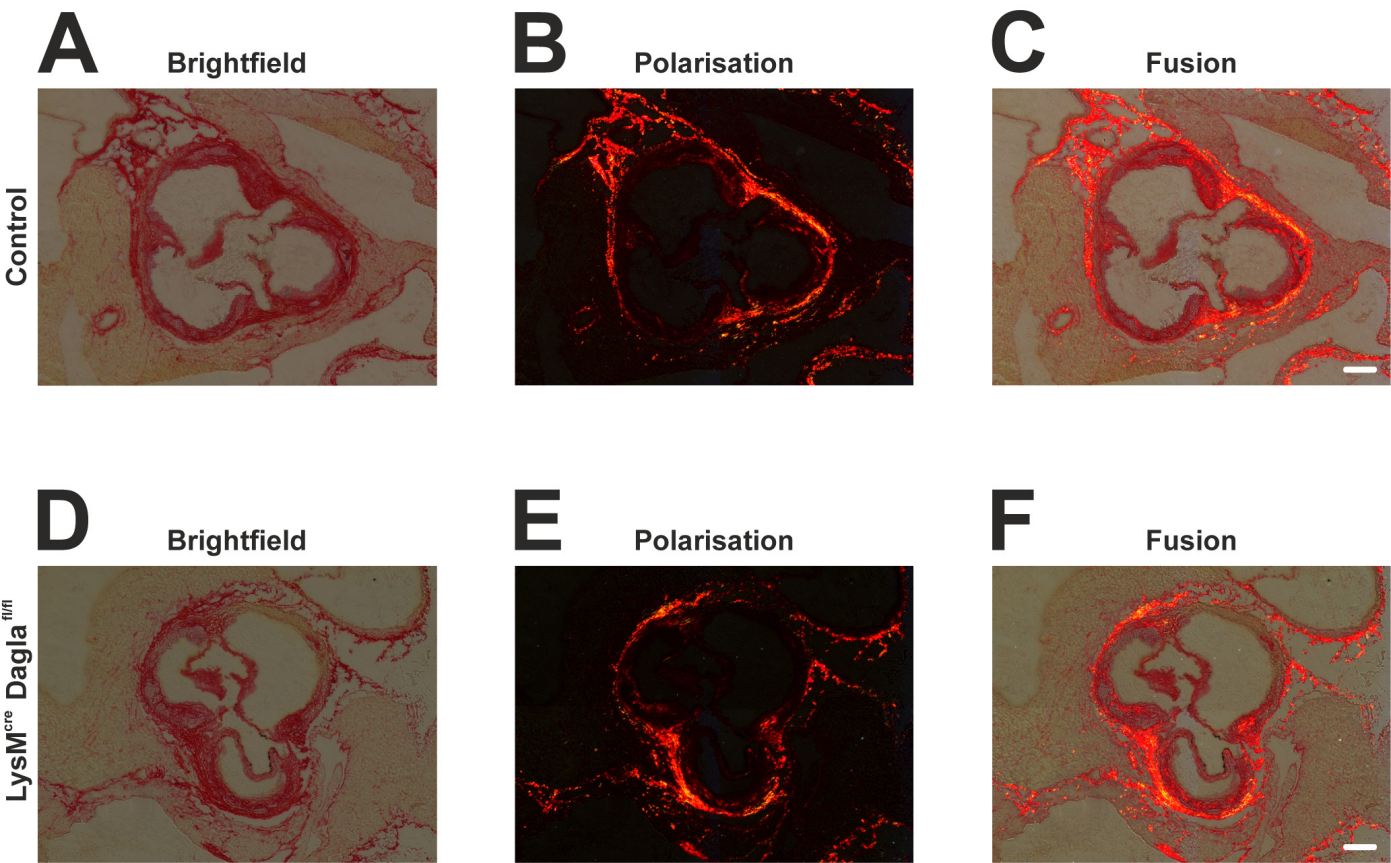
Collagen structures within the atherosclerotic aortic sinus. Frozen sections of both control mice and Dagla-deficient mice were stained using haematoxylin and direct red to visualise collagen structures. Microphotographic pictures were taken using bright field microscopy (A, D), polarisation microscopy (B, E), or a combination of both (C, F). No differences were seen in amount and distribution of collagen fibers between control mice (A-C) and Dagla-deficient mice (D-F). Scale bar, 200 μm. Cre, cyclisation recombination; Dagla, diacylglycerol lipase α; fl, flanked by loxP.

Collagen fibres were stained by Picro Sirius Red staining. The amount and distribution of collagen fibres was not significantly altered by genetic disruption of Dagla (Fig 2).

**Fig 3:**
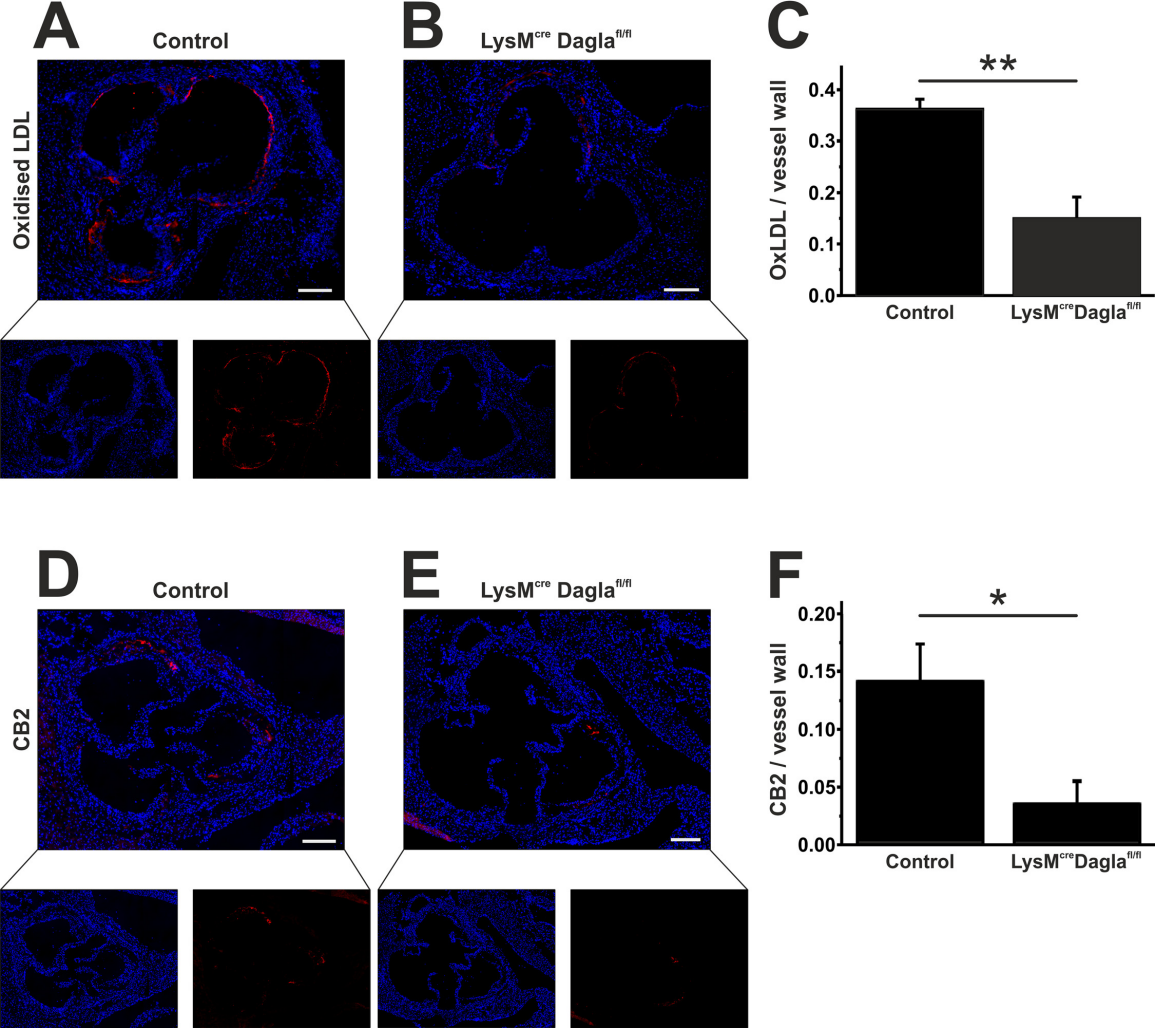
Assessment of oxLDL and CB2 within atherosclerotic lesions. Frozen sections through the aortic sinus of both control mice and Dagla-deficient mice were stained for oxLDL. OxLDL formation was predominant in the endothelium and within atherosclerotic plaques (A, B). OxLDL formation was significantly diminished in Dagla-deficient mice (C). Staining for CB2 revealed a significantly reduced CB2 expression within the aortic sinus (D-F). Data are presented as mean ± standard error of the mean; n = 3–5; *, p < 0.05; **, p < 0.01. Scale bar, 200 μm. CB2, cannabinoid receptor 2; Cre, cyclisation recombination; Dagla, diacylglycerol lipase α; fl, flanked by loxP; LDL, low-density lipoprotein; LysM, lysozyme 2 gene.

OxLDL was quantified in histological sections through the aortic sinus. As depicted in Fig 3 A-C, the endothelium strongly stained for oxLDL as well as atheromatous plaque areas. OxLDL positive areas were substantially diminished in sections of Dagla-deficient mice compared to Dagla-unimpaired controls (0.36 ± 0.02 vs. 0.15 ± 0.04, n = 4–5, p = 0.004).

CB2 expression colocated nicely with CD68 expression of neighbouring sections and was predominant in the areas of plaqe. As reported for CD68, CB2 expression was significantly reduced in mice with an impaired 2-AG biosynthesis (0.14 ± 0.03 vs. 0.04 ± 0.02, n = 3, p = 0.048; Fig 3D–3F).

**Fig 4:**
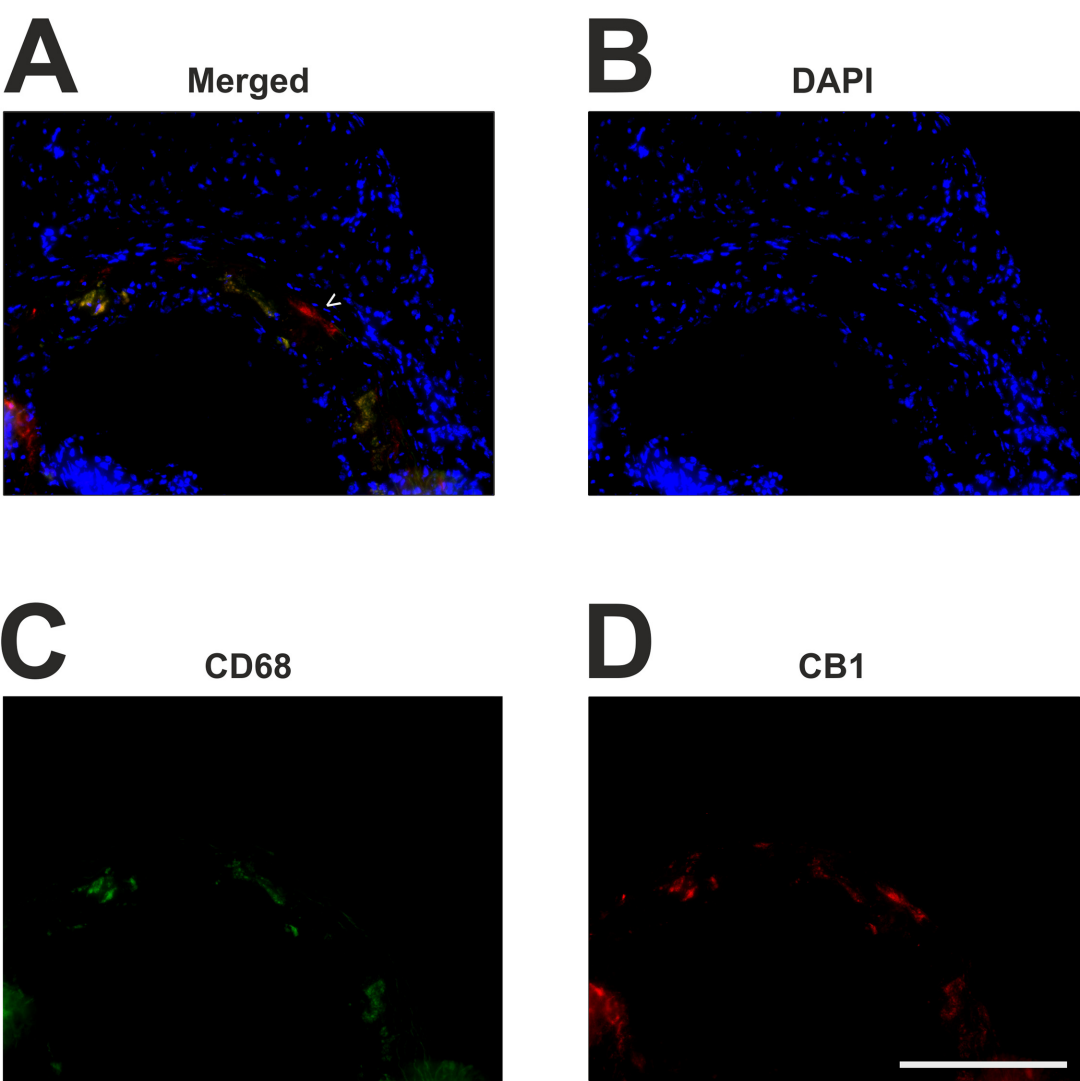
Immunofluorescent costaining of CB1 and CD68. Frozen sections through the aortic sinus were stained for CD68 and CB1 using Cy2 (green, C) and Cy3 (red, D) fluorochroms. Nuclei are stained with DAPI (blue, B). Lesional CD68-positive monocytes and macrophages (C) are the main contributors to lesional CB1 receptor expression (D) Very few cells express CB1 which do not express CD68 (arrow head, A). Scale bar, 200 μm. CB1, cannabinoid receptor 1; CD68, cluster of differentiation 68; DAPI, 2-(4-amidinophenyl)-6-indolecarbamidine dihydrochloride.

As depicted in Fig 4, few cells stained positively for CB1 receptor expression. The signal from CB1 staining was fainter compared to CB2 staining. This matches the fact, that CB2-receptor expression is more pronounced on immune cells than CB1-receptor expression, which is in turn predominant in the central nervous system. Microfotographic pictures at a higher magnification demonstrated that CB1 receptor expression was detected on but not restricted to nearly all CD68 positive cells. However, CD68 positive monocytes and macrophages appear to be the main contributors to lesional CB1-receptor expression. This finding was irrespective of Dagla deficiency in both groups.

We finally quantified T-cells within the atherosclerotic vessel wall by staining CD3 in frozen sections of the aortic sinus. The amount of T-cells was not altered by Dagla deletion (0.2 ± 0.08% vs. 0.5 ± 0.03%, n = 4–5, p = 0.25).

### 3.5. Circulating inflammatory cells

Dagla-deficient mice showed a significantly reduced accumulation of neutrophil granulocytes and macrophages in the atherosclerotic vessel wall, which might be due to an altered leukopoiesis or an increased liberation of leukocytes from the bone marrow. To rule out these systemic effects in the chimeric knockout mice, we performed flow cytometry analyses of components of the innate and adaptive immune response of bone marrow and peripheral blood. Bone marrow and peripheral blood samples were stained for CD19, CD3, CD11b, Ly6C and Ly6G and quantified by flow cytometry. No differences were seen in the expression of these surface antigenes on immune cells from Dagla-deficient mice and controls. Triple staining, using CD11b, Ly6C and Ly6G antibodies, was performed to analyse leukocyte subpopulations, which did not differ significantly between both groups (Table 2).

**Table 2:** Flow cytometry analyses of bone marrow and peripheral blood.

**A**			
**Antigen**	**Control**	**LysM**^**cre**^ **Dagla**^**fl/fl**^	**p**
CD19 (B-cells) [% of nucleated cells]	20.75 ± 4.04	16.11 ± 1.17	0.2852
CD11b^+^Ly6C^+^Ly6G^+^ (neutrophils) [% of nucleated cells]	56.05 ± 4.61	59.24 ± 0.95	0.5068
CD11b^+^Ly6C^+^Ly6G^-^ (monocytes) [% of nucleated cells]	4.74 ± 0.46	4.79 ± 0.49	0.9319
**B**			
CD19 (B-cells) [% of nucleated cells]	40.70 ± 2.75	43.82 ± 3.23	0.6055
CD3 (T-cells) [% of nucleated cells]	7.19 ± 0.63	6.22 ± 0.34	0.1920
CD11b^+^Ly6C^+^Ly6G^+^ (neutrophils) [% of nucleated cells]	16.51 ± 1.90	17.32 ± 1.79	0.7595
CD11b^+^Ly6C^+^Ly6G^-^ (monocytes) [% of nucleated cells]	6.84 ± 0.61	6.59 ± 0.58	0.7696

Bone marrow (A) and peripheral blood leukocytes (B) were stained for CD3 (FITC-conjugated), CD19 (PE-conjugated), and CD11b (APC-conjugated) or CD11b (PE-conjugated), Ly6C (FITC-conjugated), and Ly6G (APC-conjugated) respectively. Prevalence of these surface markers within a specified leukocyte gate was determined after measuring 200,000 counts (bone marrow) or 100,000 counts (peripheral blood) during FACS analysis. Values represent positively stained cells as a percentages of all nucleated cells. Data are presented as mean ± standard error of the mean; n = 10. CD, cluster of differentiation; cre, cyclisation recombination; Dagla, diacylglycerol lipase α; fl, flanked by loxP; Ly6C, lymphocyte antigen 6C; Ly6G, lymphocyte antigen 6G; LysM, lysozyme 2 gene.

### 3.6. Plasma levels of inflammatory cytokines

MCP-1 expression levels in plasma samples did not differ significantly between the two groups (Controls: 69 ± 27 pg/ml vs. LysM^wt/cre^ Dagla^fl/fl^: 568 ± 440 pg/ml, n = 5, p = 0.37).

Likewise, plasma expression levels of IL-10 showed no significant differences between the two groups (Controls: 472 ± 249 pg/ml vs. LysM^wt/cre^ Dagla^fl/fl^: 133 ± 34 pg/ml, n = 5, p = 0.25). Plasma levels of IFN-γ consistently lay below the detection threshold in both groups.

### 3.7. Liquid chromatography-multiple reaction monitoring

**Fig 5:**
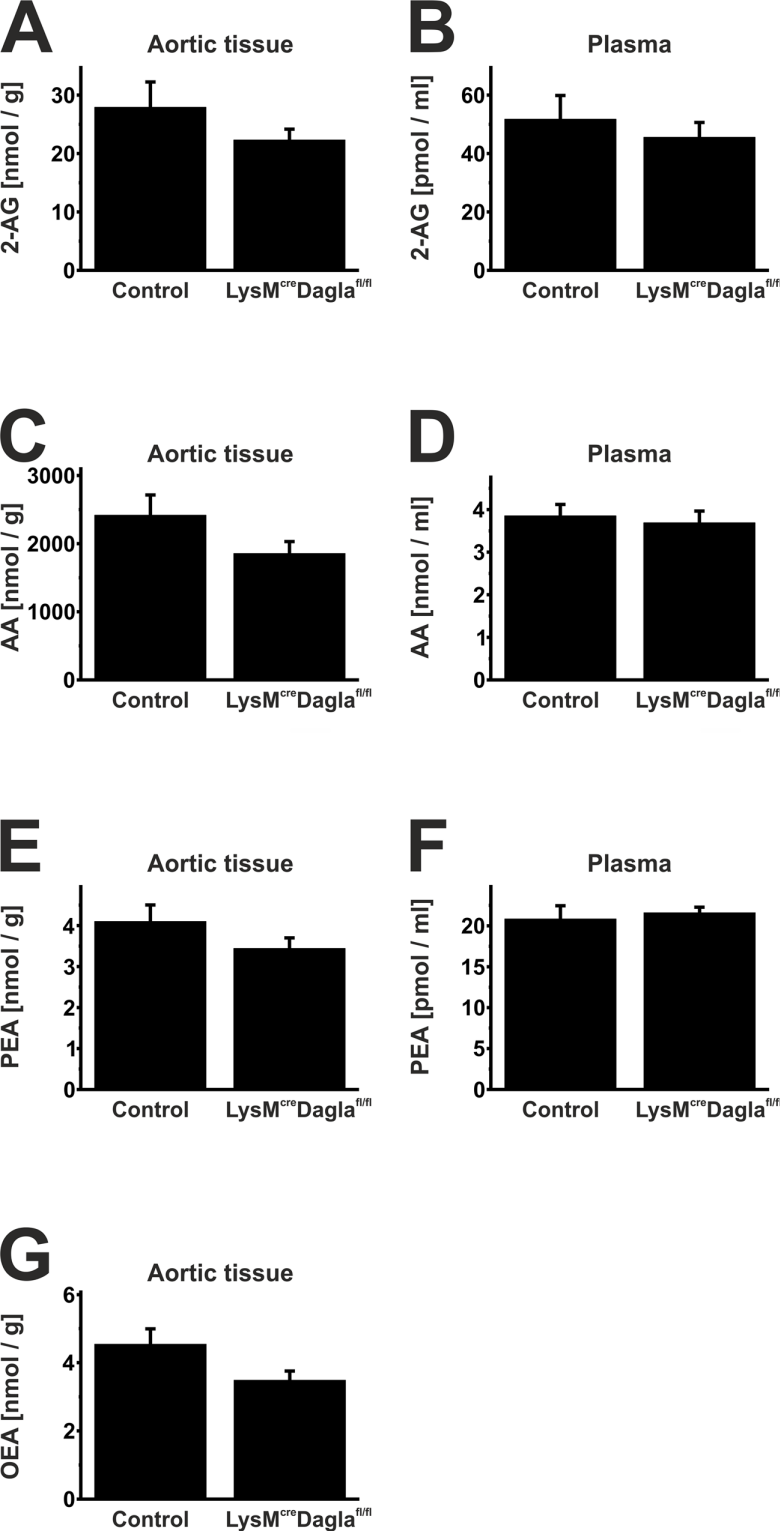
Endocannabinoid levels in plasma and aortic tissue. Endocannabinoid levels were measured in plasma and aortic tissue of both control mice and Dagla-deficient mice by liquid chromatography-multiple reaction monitoring. Dagla-deficient mice showed a mild reduction in 2-AG levels (A, B). Levels of AA, PEA and OEA did not differ significantly between both groups (C-G). Data are presented as mean ± standard error of the mean; n = 10; p > 0.05. 2-AG, 2-arachidonoylglycerol; AA, arachidonic acid; cre, cyclisation recombination; Dagla, diacylglycerol lipase α; fl, flanked by loxP; OEA, oleylethanolamide; PEA, palmitoylethanolamide.

Endocannabinoid levels were measured in plasma and aortic tissue of chimeric ApoE^-/-^ LysM^wt/cre^ Dagla^fl/fl^ mice and control mice (Fig 5). Plasma samples showed no significant differences in endocannabinoid levels, even though plasma levels of 2-AG tended to be reduced in chimeric Dagla knockout mice (51.77 ± 8.11 vs. 45.57 ± 5.05 pmol/ml, n = 10, p = 0.52; Fig 5). Accordingly, a mild reduction of 2-AG was detected in aortic tissue of Dagla-deficient mice, compared to mice with an intact 2-AG synthesis, which did not reach satistical significance (27.92 ± 4.34 nmol/g vs. 22.32 ± 1.73 nmol/g, n = 10, p = 0.25). 2-AG plasma levels of age-matched wildtype mice in the absence of an atherogenic diet yielded 49.49 ± 1.06 pmol/ml (n = 3). In the aortic tissue of wildtype mice, 2-AG levels were 26.98 ± 6.03 nmol/g (n = 8). Hence, both plasma and aortic tissue 2-AG levels of wildtype mice did not differ significantly from the two other groups (Plasma: p_wt vs. control_ = 0.88; p_wt vs. Dagla knockout_ = 0.69. Aortic tissue: p_wt vs. control_ = 0.90; p_wt vs. Dagla knockout_ = 0.43).

## Discussion

The endocannabinoid system is an integral regulator of vascular inflammation and atherogenesis (reviewed by [18]); [14]. While the role of anandamide in atherosclerosis has already been focus for several mechanistic studies, no basic research study has examinined the causal impact of 2-AG on vascular inflammation and atherogenesis in vivo yet. In the present study, we characterised the significance of 2-AG in an atherosclerosis prone animal model in which the myeloid biosynthesis of 2-AG has been genetically disrupted. Following a high cholesterol diet for 8 weeks, this Dagla-deficient mouse model showed a significantly reduced plaque burden compared to control mice with an intact 2-AG biosynthesis. This reduction in plaque size was accompanied by a significantly diminished accumulation of both macrophages and neutrophil granulocytes in the atherosclerotic vessel wall. Meanwhile, the prevalence of T-cells within the vessel wall was unaltered in Dagla deficient mice. These findings indicate, that 2-AG exerts its proinflammatory functions by facilitating accumulation of cells of the innate immune system in the vessel wall and thereby augments atherosclerotic lesion formation. The importance of innate immune cells in atherogenesis has repeatedly been demonstrated before (reviewed in [19]).

Next, CB2 receptor expression was quantified by immunofluorescence staining. CB2 receptor expression was seen to be significantly reduced in Dagla-deficient mice. Moreover, we found a robust colocalisiation of CB2 and CD68 positive areas within the atherosclerotic vessel wall. Since macrophages display the highest CB receptor expression among immune cells, it seems likely that the reduced levels of CB2 expression in atherosclerotic lesions of Dagla-deficient mice are due to the reduced amount of inflitrating macrophages [20].

Furthermore, oxLDL formation was found significantly reduced in Dagla-deficient mice. One possible explanation for this might be that 2-AG influences cholesterol uptake and efflux in lesional macrophages. Interestingly, the synthetic cannabinoid Win55.212–2 has already been demonstrated to increase the expression of scavenger receptor CD36 (oxLDL uptake) in murine primary peritoneal macrophages. Additionally, Win55.212–2 causes a decrease in the expression of ATP-binding cassette protein ABCA1 which is responsible for oxLDL efflux [21]. One may speculate that similar mechanisms might be triggered by endocannabinoids and that their lack might cause the phenotype observed in the present study.

Extensive FACS analyses were performed, to study whether changes in leukopoiesis or changes in leukocyte levels in peripheral blood account for altered neutrophil and macrophage invasion into the vessel wall. However, no changes were seen in the prevalence of distinct surface antigens in bone marrow and peripheral blood samples of Dagla-deficient mice compared to controls. These findings suggest that the local atherosclerotic microenvironment and/or the interaction with susceptible inflammatory cells such as monocytes is responsible for the observed changes in the prevalence of immune cells in atherosclerotic lesions, rather than augmented numbers of circulating inflammatory cells. These observations match the fact that endocannabinoids mainly exert autocrine and paracrine effects (reviewed in [22]). The present study suggests that myeloid cells might ameliorate their own migration towards atherosclerotic lesions by secretion of 2-AG and that accordingly disruption of 2-AG synthesis reduces vascular inflammation. This hypothesis is very intriguing since, albeit subject to controversial discussions, several studies indicate a proinflammatory role of 2-AG in various in vitro models. Stimulation of human HL-60 monocytes with 2-AG, for example, leads to an increased mRNA expression of proinflammatory cytokines IL-8, TNF-α and MCP-1 [8]. Accordingly, 2-AG has been reported to induce migration of HL-60 derived macrophage-like cells [23] and human monocytes [7, 14]. In the present study, however, conditional knockout mice did not display altered systemic plasma levels of inflammatory cytokines. Probably, conditional knockout of 2-AG synthesising enzyme Dagla is insufficient to yield systemically altered cytokine levels. Though, concentrations of inflammatory cytokines may vary locally and thereby contribute to a pro-inflammatory microenvironment which might accelerate atherogenesis. Data from both atherosclerotic animal models and human studies suggest that proinflammatory effects of 2-AG might be mediated by the CB1 receptor in vivo, whose blockade promotes atheroprotection [24] and whose expression is elevated in patients suffering from coronary artery disease [15]. Clearly, further studies using tissue-specific impairment of either cannabinoid receptor will be necessary to understand their roles in atherogenesis and the contribution of 2-AG to vascular inflammation.

LC-MRM analyses of plasma samples showed no significant differences between both groups, while 2-AG levels of Dagla-deficient mice tended to be reduced in plasma and aortic tissue, compared to controls. The relatively small reduction in 2-AG levels of both plasma and aortic tissue of Dagla-deficient animals may be explained by the little contribution of myeloid endocannabinoid synthesis to the total endocannabinoid content of plasma and aortic tissue. Another possible explanation might be, that diacylglycerol lipase β, whose relevance to the 2-AG synthesis in macrophages has been reported, also contributes to 2-AG contents of plasma and aortic tissue [25]. Furthermore, levels of AA, OEA and PEA tended to be reduced in Dagla-deficient mice, which did not reach statistical significance. Reduction in AA levels following Dagla knockout is understandable, keeping in mind that 2-AG contributes to the arachidonic acid pool by hydrolysis. Further experimental approaches targeting different components of the endocannabinoid system are needed to fully understand the underlying interactions of endocannabinoids and lipid molecules.

The present study is the first to describe a causal connection between myeloid DAGL-α expression and vascular inflammation and atherogenesis. This finding is particularly intriguing, since 2-AG, which is synthesised by DAGL, is the most abundantly expressed endocannabinoid in the atherosclerotic aortic vessel wall [5]. Additionally, patients suffering from coronary artery disease exhibit significantly increased plasma levels of 2-AG [15] which underlines potentially beneficial effects of a reduced 2-AG biosynthesis in clinical practice. A better understanding of the endocannabinoid system in atherogenesis may help to develop novel therapeutic strategies against atherosclerosis.

## Abbreviations

2-AG: 2-arachidonoylglycerol
AA: Arachidonic acid
AEA: N-arachidonoylethanolamide
CB1: Cannabinoid receptor 1
CB2: Cannabinoid receptor 2
Dagla: Diacylglycerol lipase α
Daglb: Diacylglycerol lipase β
eCBs: Endocannabinoids
ELISA: Enzyme-linked immunosorbent assay
LC-MRM: Liquid chromatography-multiple reaction monitoring
MAGL: Monoacylglycerol lipase
NGS: Normal goat serum
OEA: Oleylethanolamide
OxLDL: Oxidised low-density lipoprotein
PBS: Phosphate buffered saline
PCR: Polymerase chain reaction
PEA: Palmitoylethanolamide
